# Synthetical Chemistry and Its Relation to the Materia Medica of the Future

**Published:** 1894-06

**Authors:** Frank S. Hough


### Synthetical Chemistry and Its Relation to the Materia Medica of the Future.

BY FRANK S. HOUGH, M.D.

Lecturer on Chemistry in the Michigan College of Medicine and Surgery, Detroit, Michigan.

There are two terms expressive of two processes exactly opposite in their nature, but which have operated together as co-partners in the development and gradual evolution of the sciences, especially in relation to the sciences of chemistry and materia medica. These two technical terms are analysis and synthesis.

For years, and until recently, materia medica was studied and applied more from a purely clinical than a chemical standpoint. Through the manner in which the drugs seemed to impress themselves upon the human organization, and through the selective action they seemed to possess towards certain physiological functions and processes, in experimentation on vast numbers of cases there was built up the great and massive structure of materia medica. Physicians learned to prescribe certain drugs for certain pathological conditions and derangements of functional activity, because experience and the written and verbal evidence and close observation taught them that these drugs brought about cure or amelioration of the symptoms. This was the manner in which the great compilation of medical substances was effected and the voluminous treatises and works on materia medica were collected and interwoven into that branch of medical science.

With the progress of medicine towards exactitude, and the impetus given to it by closer methods of examination into the causes of its effects in all its various branches, physicians began to cultivate a chemical knowledge of the hundreds and hundreds of substances employed in the application of the healing art, till finally they began to inquire into the composition and minute anatomy of the drugs and to search out reasons for the diversity of actions presented by the various drugs. Analysis then took a hand in the work, and threw its search-light into the shadowy recesses, and sought to dispel the mists. To say that the results were signal is but to voice a platitude. Chemists began to analyze everything used as therapeutics, and they found that drugs regarded as simple by our medical ancestors exhibited a chemical structure so complex as to almost baffle their efforts, but time and perseverence served to untangle the intricacies and bring order out of chaos. Side by side with materia medica, then, was built up the enduring monument of organic chemistry, and to its achievements and the work of its exponents belong all the science —all that is exact and scientific.

Then, and not till then, was it determined that medical substances having more than one action, and hence more than one use, contained in their chemical anatomy more than one group or combination of elements. It was likewise learned that a great many of the more potent drugs contained in their chemical interior, active principles responsible for the therapeutical phenomena brought out in their action, and that the active principle of a single drug constituted only a small portion of the crude drug. It was also demonstrated that many of the drugs were masses composed of groupings of several of these powerful active principles, and that they owed their peculiarities and characteristics to aggregations of these potent factors. To illustrate, I have but to mention the drug opium. At first blush it would seem but a simple medicine, but when you consider that it is a bulk in whose composition there are eighteen or more distinct and separate alkaloids, each having its own chemical formulæ and its individual physiological effect, you will cease to regard it in such an elementary light. Even the sum total of the alkaloidal bases in it does not account for its entire body, as, besides these active principles, there are other organic substances, or groupings of elements that contribute much towards its construction.

Opium is but one good example of the complexity of formulæ concerned in the make-up of many substances belonging to materia medica. Numerous other substances are equally as complex.

Chemical analysis has added tenfold to the armamentarium of the physician by splitting up compound substances into component simpler compounds, and thereby multiplying each drug so treated, but this is not its most important work. By resolving complicated formulæ into simpler formulæ it has, by inspecting these formulæ, laid the foundation for the new chemical materia medica. To explain:—These simpler groupings of ultimate elements are closely analyzed and it has been discovered that when certain elements exist in combination, with certain other elements, and when administered, they produce certain effects. By close study of the elements occurring in combination in certain proportions to each other, it has been made possible to artificially bring together these same elements in similar proportions, and to make medicines in the laboratory which are as effective as the natural products furnished by analytical decomposition of nature's remedies. What may be more astonishing, but is nevertheless true, is the fact that in many instances these synthetical, or artificial compounds can be made much more active than the natural compounds resulting from the decomposition of natural substances. Chemists, who are also learned in medicine by clinical experience find that a combination in a great measure owes its utility to one or two of its composing elements, and when they reproduce a similar compound, by synthetical process they naturally increase, if possible, the element responsible for the use of the compound. To give you an illustration of this principle, put into practice, we will study iodoform. Iodoform is a simple compound of the organic base formyl and iodine and has a formula expressed, $CHI_3$. The usefulness of iodoform depends entirely upon the iodine. The iodine alone exerts the antiseptic power. It was desired to have a compound containing iodine in such an amount and in such a loose condition of union that when applied to the tissue the iodine would be liberated gradually and exert its antiseptic power continuously. Had the chemist made his compound so that the iodine was united with another base in which the union was more tenacious the iodine would not be let loose to do its work. Did the compound contain less iodine, the condition of affairs would not favor its liberation. Pure iodine would be too powerful and would be out of the question. Here is where your educated chemist had a chance to exercise his ingenuity, and by thought he produced the ideal compound—a compound which would when applied to the tissues allow enough iodine to volatilize continuously and exert its salutary action, and yet volatilize only fast enough to do good without irritating the tissues too much by furnishing the iodine in too large a quantity.

By this time it is patent to you that by chemical analysis the chemist has discovered the very fountain head. He has found that complex structures owe their virtues to simpler compounds of elements, and that in many of these lesser combinations the essence is in one single element. So much for analysis, which while being the real foundation of the edifice is not the structure itself. Synthesis, synthetical chemistry, is the giant that is revolutionizing materia medica and furnishing the hundreds and hundreds of new substances all of which are chemical products, pure and simple, and are being manufactured to accomplish remedial purposes and directed against all the pathological conditions the diagnotician can detect. Most of the inorganic salts used in the practice of medicine, are the foot-prints of this Colossus in the past, the coal-tar derivatives are its present progeny, and who knows what an infinite variety may be its future product? Our wildest speculations would not serve to measure the onward march of progress this great factor, synthetical chemistry, is making and will make. Thousands of chemical drugs, representing a scale of graduations too minutely fine to imagine, are to be added to materia medica.

Chemistry, as a branch of medical education, has been relegated to the darkest corner in the college curriculum of most institutions; but the time has arrived when a thorough and accurate knowledge of chemistry is absolutely essential to an understanding of drugs and their uses. Materia medica is to be enormously developed, but mainly through the development of organic chemistry. * * * * *—*Detroit Emergency Report.*

